# Involvement of Macrophages in the Pathogenesis of Transmissible Spongiform Encephalopathies

**DOI:** 10.1080/10446670290030981

**Published:** 2002-03

**Authors:** Vincent Beringue, Patrick Couvreur, Dominique Dormont

**Affiliations:** 1 CEA, Service de Neurovirologie DRM/DSV CRSSA Fontenay aux Roses France; 2 Laboratoire de Physico-Chimie Pharmacotechnie, Biopharmacie, UMR CNRS 8612 Facult' de Pharmacie Chatenay-Malabry France

## Abstract

Although transmissible spongiform encephalopathies (TSE) or prion diseases are neurodegenerative disorders, the immune system is also involved, at least in the early stages of their pathogenesis. Extensive studies have focused on cells targeted by the TSE agent for its replication but few on the possible involvement of macrophages in its clearance, as in more conventional diseases. This review summarises some of the experiments aimed at demonstrating a role for macrophages in TSE and presents the application to TSE of the macrophage "suicide" technique, which has been used to clarify the implication of these cells in the early steps of TSE pathogenesis.


*Developmental Immunology*, 2002 Vol. 9 (1), pp. 19–27

Taylor & Francis healthsciences

### Involvement of Macrophages in the Pathogenesis of Transmissible Spongiform Encephalopathies

VINCENT BERINGUE[a,*], PATRICK COUVREUR[b] and DOMINIQUE DORMONT[a]

[a]*CEA, Service de Neurovirologie, DRM/DSV, CRSSA, Fontenay aux Roses, France;* [b]*Laboratoire de Physico-Chimie, Pharmacotechnie, Biopharmacie, UMR CNRS 8612, Faculté de Pharmacie, Chatenay-Malabry, France*

Although transmissible spongiform encephalopathies (TSE) or prion diseases are neurodegenerative disorders, the immune system is also involved, at least in the early stages of their pathogenesis. Extensive studies have focused on cells targeted by the TSE agent for its replication but few on the possible involvement of macrophages in its clearance, as in more conventional diseases. This review summarises some of the experiments aimed at demonstrating a role for macrophages in TSE and presents the application to TSE of the macrophage "suicide" technique, which has been used to clarify the implication of these cells in the early steps of TSE pathogenesis.

*Keywords*: PrP; Scrapie; Prion; Macrophage; Clodronate; Spleen

### INTRODUCTION

Transmissible spongiform encephalopathies (TSE) or prion diseases are a group of fatal neurodegenerative disorders, including natural scrapie in sheep and goats, bovine spongiform encephalopathy (BSE) in cattle and Creutzfeldt–Jakob disease (CJD) in humans. CJD itself can be classified in three sub-categories: sporadic, inherited (associated with coding mutations in the prion protein gene PRNP) or infectious (encompassing contamination with CJD agent during medical interventions). Variant CJD (vCJD) is the most recent recognised form of infectious CJD and thought to originate from BSE (for review see, Collinge, 1999). TSE are characterized by the accumulation of a partially protease-resistant isoform (PrPres) of the host encoded prion protein ($PrP^c$) in the central nervous system (Prusiner, 1982). PrPres is mainly associated with TSE agent infectivity; but whether or not this abnormal protein constitutes its sole component remains controversial (Caughey and Chesebro, 1997; Lasmezas *et al.*, 1997; Prusiner *et al.*, 1998).

Most of our knowledge of TSE pathogenesis has been established using experimental rodent models adapted from natural disease (Kimberlin and Walker, 1988). Studies after peripheral infection are particularly important, as it represents the major route of contamination for many natural TSE such as BSE, iatrogenic CJD related to human growth hormone contamination and probably vCJD. After intraperitoneal infection, the lymphoreticular system (LRS) constitutes the first target for TSE agent replication: in murine models of scrapie and BSE, infectivity (Kimberlin and Walker, 1988) and PrPres are rapidly detected in the spleen and soon after, in Peyer's patches, lymph nodes, thymus and pancreas (Grathwohl *et al.*, 1996; Beringue *et al.*, 1999a; Maignien *et al.*, 1999). After oral infection with murine BSE or scrapie, PrPres is detectable first in Peyer's patches and then in lymph nodes and in the spleen (Maignien *et al.*, 1999). This peripheral contamination is observed long before neuroinvasion occurs (Kimberlin and Walker, 1988; Beringue *et al.*, 1999a; Maignien *et al.*, 1999). Tissues of the immune system are similarly infectious in natural scrapie (Hadlow *et al.*, 1982) and more strikingly accumulates PrPres in vCJD (Hill *et al.*, 1999), unlike other forms of CJD (Kitamoto *et al.*, 1989; Brown *et al.*, 1994). The transport of the TSE agent from the LRS to the central nervous system most probably takes place via autonomic nerves linking LRS organs to the thoracic spinal cord (Kimberlin and Walker, 1988; 1989; Beekes *et al.*, 1996) or via the vagus nerve (Beekes *et al.*, 1998).

The nature of immune cells involved in TSE agent replication in the LRS is not clearly known. Replication does not exclusively occur in T cells since both thymectomized mice, *nude* mice and transgenic mice lacking functional T cells are fully susceptible to experimental scrapie infection, after peripheral inoculation (Fraser and Dickinson, 1978; Mohri *et al.*, 1987; Klein *et al.*, 1997). Moreover, $PrP^c$ expression restricted

*Corresponding author. Address: Institut National de la Recherche Agronomique, Virologie and Immunologie Moléculaires, 78 352 Jouy en Josas Cedex, France. Tel.: +33-1-34-65-26-16. Fax: +33-1-34-65-26-21. E-mail: beringue@jouy.inra.fr

ISSN 1044-6672 print © 2002 Taylor & Francis Ltd
DOI: 10.1080/10446670290030981

TABLE I Reduction in scrapie infectivity after *in vitro* mouse peritoneal macrophages incubation with mouse scrapie strain ME7 (from Carp and Callahan, 1982)

| Recipient mice* inoculation with: | Scrapie incubation period (days ± s.e.m.) after *in vitro* culture for: | | | |
|---|---|---|---|---|
| | 2 h | 4–5 days | 14 days | 28 days |
| Without wash | | | | |
| ME7† | 161 ± 2 | 169 ± 2 | | |
| ME7 + macrophages† | 163 ± 2 | 177 ± 2‡ | | |
| With wash | | | | |
| ME7 | 165 ± 1 | 168 ± 1 | | |
| ME7 + macrophages | 170 ± 3 | 183 ± 3‡ | 263 ± 15‡ | |
| ME7 + macrophages | 203 ± 7 | | | 332 ± 48‡ |

Macrophages were exposed to 1% scrapie brain homogenate for 2 h at 37°C. They were then either washed or not, before extended culture until 28 days. Scrapie infectivity associated to the culture was assessed by measuring incubation period in recipient mice infected with the latter.
* Five to six mice were inoculated. In each experiment, all developed scrapie.
† Mean of five differents experiments.
‡ Statistically significant ($p < 0.01$).

to T lymphocytes did not allow scrapie agent replication in the LRS after intraperitoneal infection (Raeber *et al.*, 1999a). However, splenic T cells have been shown to contain substantial amounts of scrapie infectivity (Lavelle *et al.*, 1972; Raeber *et al.*, 1999b); but only when they express $PrP^c$ at their surface (Raeber *et al.*, 1999b). Whole body irradiation and splenocytes fractionation have suggested that the cells supporting scrapie agent replication are non-dividing stromal cells (Clarke and Kimberlin, 1984; Fraser and Farquhar, 1987). Furthermore, PrPres accumulates in large amounts in splenic follicular dendritic cells (FDC) of scrapie-infected mice (McBride *et al.*, 1992; Muramoto *et al.*, 1993). Hence, TSE agent replication may occur specifically within FDC. The presence of mature B cells is necessary for scrapie propagation, whether or not they express $PrP^c$ (Klein *et al.*, 1997; 1998) and splenic B lymphocytes are associated with scrapie infectivity only when they express $PrP^c$ (Raeber *et al.*, 1999b). This can be most readily explained by a requirement for B cells in FDC maturation (Fu *et al.*, 1998; Gonzalez *et al.*, 1998). Whatever the relative importance of individual cell types, a partly or totally functional immune system is required for scrapie propagation from the periphery to the central nervous system after intraperitoneal infection, as the disease is delayed in splenectomized mice (Fraser and Dickinson, 1978) and did not or occur rarely in a panel of immunodeficient mice with deficits in B/T, B lymphocytes and FDC development, such as SCID mice, $Rag^{0/0}$ or μMT mice (O'Rourke *et al.*, 1994; Lasmezas *et al.*, 1996; Klein *et al.*, 1997; 1998; Beringue *et al.*, 1999b).

The involvement of macrophages in the pathogenesis of experimental TSE has also been studied: their phagocytic capacity could theoretically interfere with TSE agent replication and conversely they could also permit replication since they are radioresistant, express $PrP^c$ and are distributed throughout LRS tissues. This review synthesises experiments performed to define the role of macrophages.

### *IN VITRO* INTERACTION OF PERITONEAL MACROPHAGES WITH THE SCRAPIE AGENT

Carp and Callahan (1981) showed that mouse scrapie inoculum and mouse peritoneal macrophages associated *in vitro*, suggesting an interaction between macrophages and the scrapie agent, probably due to phagocytosis. They then measured infectivity associated with a culture of peritoneal macrophages, exposed during 2 h with scrapie inoculum (Carp and Callahan, 1982). Variations in infectivity were assessed by measuring scrapie incubation period in recipient mice infected with the macrophage culture (Table I and Carp and Callahan, 1982). Incubation of peritoneal macrophages during 4–5 days after exposure to scrapie homogenate increased recipient mice incubation period, suggesting that they have destroyed some infectivity, compared to mice inoculated with the 2 h incubation mixture or with the scrapie strain used (ME7) incubated without the cells (Table I). Peritoneal macrophages were also washed after the 2 h exposure with scrapie homogenate before extended incubation, to remove the amount of scrapie agent not associated with these cells. In this case, the incubation period of recipient mice inoculated with scrapie-exposed macrophages is also prolonged, even after 28 days culture (Table I). It was noteworthy that the medium removed from the macrophage culture was not infectious and that destroying macrophages by UV irradiation after the 2 h exposure to scrapie produced more infectivity (Carp and Callahan, 1982). Therefore, the decrease in infectivity observed is likely due to macrophages activity itself.

This has also been demonstrated using the PC12, a rat phaeochromocytoma cell line, which can be differentiated into neuronal-like cells, after nerve growth factor treatment (Greene and Tischler, 1976). These cells permit the replication of the 139A scrapie strain *in vitro*, without any cytopathological changes (Rubenstein *et al.*, 1984). Exposure of scrapie-infected PC12 cells to mouse peritoneal macrophages reduced the amount of infectivity associated to the culture (Rubenstein *et al.*, 1984),

TABLE II Ratio of scrapie infectivity found in adherent and non-adherent splenocytes of mice infected intracerebrally (ic) or intraperitoneally (ip) with the 139A scrapie strain (from Carp *et al.*, 1994)

| Days of post-inoculation | Route of infection | Scrapie infectivity ($LD_{50}/10^6$ cells) a | n-a | Infectivity ratio of adherent to non-adherent cells |
|---|---|---|---|---|
| 21 | ic | $6.3 \times 10^4$ | $1.7 \times 10^4$ | 3.7 |
| | ip | $2.0 \times 10^5$ | $2.1 \times 10^4$ | 9.5 |
| 49 | ic | $4.6 \times 10^4$ | $2.3 \times 10^4$ | 2.0 |
| | ip | $3.1 \times 10^5$ | $2.2 \times 10^4$ | 14 |
| 70 | ic | $2.0 \times 10^5$ | $1.2 \times 10^5$ | 1.7 |
| | ip | $3.2 \times 10^5$ | $5.3 \times 10^4$ | 6.0 |
| 102 | ic | $3.1 \times 10^5$ | $1.4 \times 10^4$ | 22 |
| | ip | $5.0 \times 10^6$ | $6.5 \times 10^3$ | 769 |
| 118 | ic | $6.9 \times 10^4$ | $9.4 \times 10^3$ | 7.3 |
| | ip | $5.0 \times 10^4$ | $2.7 \times 10^4$ | 1.8 |
| 154 | ic | nd | nd | nd |
| | ip | $4.3 \times 10^5$ | $5.1 \times 10^3$ | 84 |

Three spleens were used per analysis point. Splenocytes were fractionated into adherent (a) and non-adherent (n-a) fraction, using tissue culture dishes with grids on their culturing surfaces. Scrapie infectivity associated to the cells was measured in recipient mice by end point titration.
nd, not determined.

suggesting again that macrophages can interfere with scrapie agent replication.

These *in vitro* studies suggest, therefore, that macrophages may be able to inactivate TSE agent, probably due to their phagocytic abilities. Experiments related to infectivity and PrPres distribution in the spleen support the idea that they could be similarly be efficient *in vivo*.

### ASSOCIATION OF INFECTIVITY AND PrPres WITH SPLEEN MACROPHAGES

### Cell Fractionation of Splenocytes from Scrapie-infected Rodents

Spleen fractionation experiments in experimental mouse and hamster scrapie have been performed to measure the amounts of infectivity associated with macrophages. In C57BL/6 mice infected with the 139A scrapie strain (Carp *et al.*, 1994), infectivity was always found in higher quantities in adherent spleen cells compared to non-adherent spleen cells, during the course of the disease, either after intracerebral or intraperitoneal inoculation (Table II). These results were identical to those observed in hamsters intraperitoneally infected with the 263 K scrapie strain (Robinson and Gorham, 1990) and support the idea that splenic macrophages could carry some infectivity. However, with a different mouse strain (Compton strain), the results were opposite (Lavelle *et al.*, 1972), suggesting that the association of macrophages with infectivity may depend on the experimental model used.

### Immunodetection of PrPres and Vacuolization Associated to Spleen Macrophages

In sheep at the terminal stages of natural scrapie, granules of PrPres have been immunodetected in macrophages associated with lymphoid follicles (Van Keulen *et al.*, 1996). In experimental mouse scrapie, fibrils of PrPres were detected by immunogold electron microscopy in lysosomes of tangible body macrophages from the splenic white pulp (Jeffrey *et al.*, 2000). Finally, during the adaptation of a hamster CJD strain (SY strain) to rats which produced inflammatory host reactions, spleen macrophages showed vacuolation early after the infection, suggesting that they could be involved in incorporating and destroying the infectious agent (Manuelidis *et al.*, 1997).

These experiments suggest therefore that, *in vivo*, macrophages could retain some infectivity/PrPres associated to TSE and can be involved in eliminating TSE agent.

### PHARMACOLOGICAL MODIFICATION OF MACROPHAGES STATUS IN EXPERIMENTAL TSE

In order to specify the role of macrophages in TSE, drugs known to modify macrophage functions *in vivo* have been tested. This has been performed in various models of experimental TSE. In earlier work, we found that association of inflammatory reactions with macrophage recruitment, induced by polyacrylamide, did not modify the incubation period of mice infected with the C506M3 scrapie strain either by intracerebral or intraperitoneal route (Dormont *et al.*, 1989). Inductions had been performed before or after the scrapie infection. Also silica, which is known to block phagocytosis repeatedly failed to modify mouse scrapie infection (Kimberlin and Walker, 1990). Finally, macrophage activation induced by thioglycolate reduced scrapie infectivity (Carp and Callahan, 1985) or not (Kimberlin and Walker, 1990), among the experiments performed. Long term oral treatment with the phenylsulfone dapsone has been shown to significantly prolong the survival time of CJD (SY strain) infected rats (Manuelidis *et al.*, 1998). It is likely that dapsone acts by activating macrophages in this inflammatory-like model (Manuelidis *et al.*, 1997; 1998), similarly to its effects on HIV-1 replication in monocyte-derived macrophages (Duval *et al.*, 1997; Clayette *et al.*, 1999).

The absence of an effect on TSE with several drugs known to interfere with macrophages status seems contradictory with experiments suggesting an interaction between these cells and infectivity or PrPres *in vivo* or *in vitro*. It is noteworthy that the consequences of these drug treatments were only studied by titrating infectivity in the terminal stage of the disease and these results do not exclude treatment effects at earlier stages of the infection, prior and during early TSE agent neuroinvasion. Moreover, the fact that infectivity must vary more than 1 Log $LD_{50}$ to be statistically significant renders consequently small effects (which are observed for silica, Kimberlin and Walker, 1990) of these drugs border-line.

### APPLICATION OF THE MACROPHAGE "SUICIDE" TECHNIQUE TO SCRAPIE-INFECTED MICE

### Principle of the Technique

The macrophage "suicide" technique consists of the administration to rodents of liposome-encapsulated dichloromethylene diphosphonate ($Cl_2MDP$), also called clodronate (Van Rooijen, 1989). As clodronate (Fig. 1) is hydrophilic, it is entrapped during the formation of multilamellar liposomes and targeted more specifically to macrophages (Van Rooijen, 1989). Clodronate is then released in these cells by phospholipase action on liposomes. Although, the mechanism of action of clodronate is unknown, the drug might inactive macrophage phagocytosis by chelating $Ca^{2+}$ or metallic ions (Van Rooijen, 1991) or directly inducing apoptosis (Naito *et al.*, 1996). Macrophages from different tissues can be specifically targeted depending on the route of administration; intravenous injection of liposome-encapsulated clodronate depletes mainly spleen macrophage subsets and Kupffer cells of liver; subcutaneous injection depletes draining lymph nodes (Van Rooijen and Sanders, 1994). Generally, macrophages are depleted by a single intravenous injection of 2 mg liposome-entrapped $Cl_2MDP$ in 200 μl (Van Rooijen, 1989).

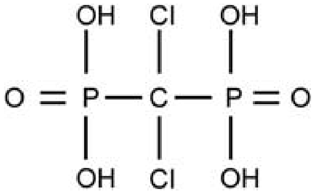

FIGURE 1 Chemical structure of dichloromethylene diphosphonate or clodronate.

a b c

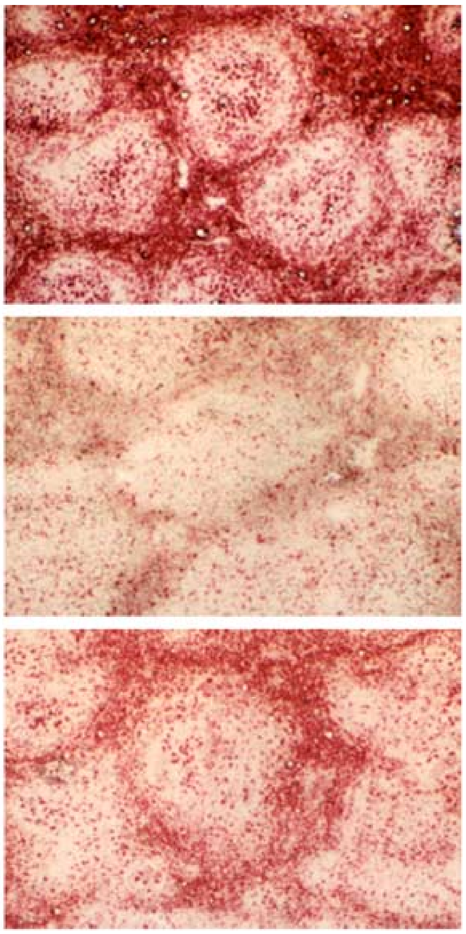

FIGURE 2 Depletion of spleen macrophages induced by liposome-encapsulated clodronate administration. Macrophage depletion was assessed on spleen sections by measuring acid-phosphatase activity 3 days (b) or 10 days (c) after intravenous liposome-encapsulated clodronate injection to mice and by comparing it to untreated or empty-liposomes treated mice (a). (×40).

### Depletion of Spleen Macrophages after Intravenous Administration of Liposome-encapsulated Clodronate

The status of spleen macrophages can be assessed by measuring acid-phosphatase activity on spleen cryostat section (Fig. 2a). Within 1 day of administration, the intravenous injection of liposome-encapsulated clodronate strongly reduces this activity (Van Rooijen and Sanders, 1994; Beringue *et al.*, 1999c) and by 3 days, elimination of all spleen macrophage subpopulations is nearly complete (Fig. 2b). Macrophages reappear on day 10 (Fig. 2c) and all macrophage subsets have fully repopulated the spleen around 1–2 months after clodronate administration (Van Rooijen *et al.*, 1989; 1990; Laman *et al.*, 1990; Beringue *et al.*, 1999c).

### Administration of Liposome-encapsulated Clodronate to Scrapie-infected Mice

As previous experiments had shown that macrophage function modifications had no effects during the late

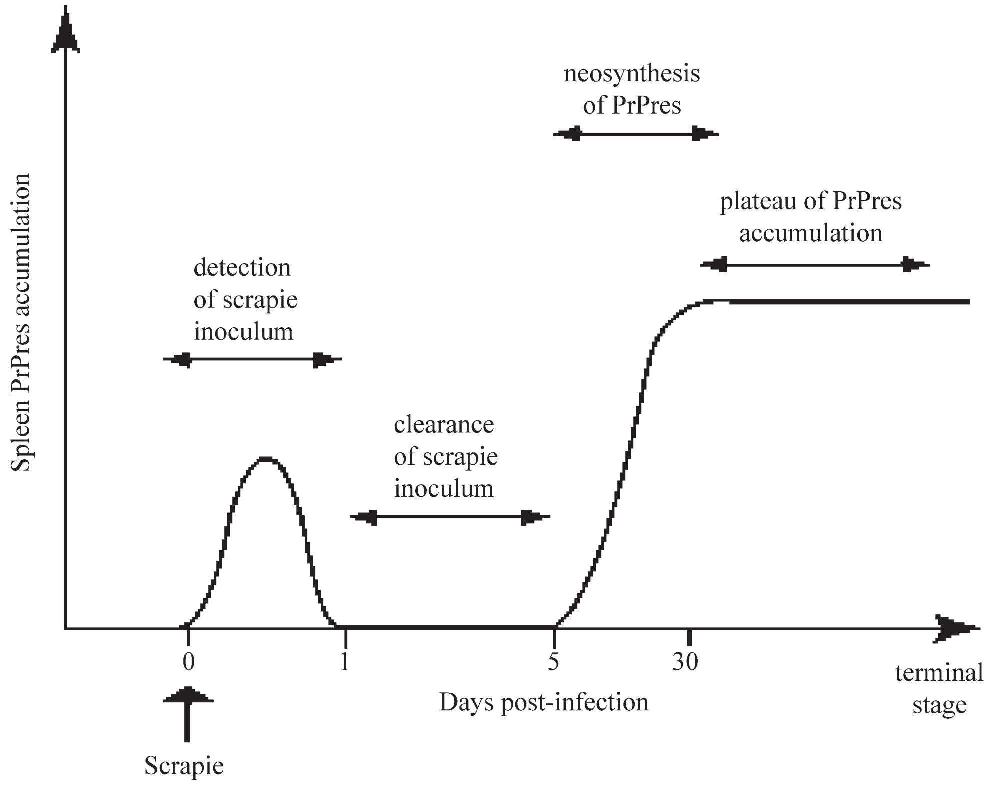

FIGURE 3 Distinct phases in PrPres accumulation in the spleen of scrapie-infected mice (from Beringue *et al.*, 1999a). C57BL/6 mice were infected with 100–200 μl of a 1–2% brain homogenate of terminally ill animals, by the intraperitoneal route. PrPres was quantified from the day of inoculation (day 0) until the terminal stage of the disease (260–280 days post-inoculation).

phases of the disease (see above), we use the macrophage "suicide" technique to study the role of macrophages during early scrapie pathogenesis in the spleen. PrPres accumulation was used as the most sensitive and specific molecular hallmark of mouse scrapie agent as it has been shown to vary significantly even when variation in infectivity was not significant, such as for the effects of the polyene antibiotic amphotericin B (Adjou *et al.*, 1999).

In C57BL/6 mice infected intraperitoneally with the C506M3 scrapie strain, the kinetics of PrPres accumulation in the spleen shows four distinct phases (Beringue *et al.*, 1999a); (i) the almost immediate detection of PrPres associated with the scrapie inoculum, (ii) a phase where the scrapie inoculum has been dispersed/cleared, where PrPres cannot be detected, from 1 to 4 days post-inoculation (dpi), (iii) the onset of the replication phase from 5 dpi, as newly synthesized PrPres accumulates in the spleen, (iv) a plateau of spleen PrPres accumulation 1–2 months after scrapie infection until the terminal stage of the disease (Fig. 3). The consequences of liposome-encapsulated $Cl_2MDP$ administration were studied on these different phases, to examine a possible scavenger role of macrophages in the early steps of scrapie pathogenesis in the spleen.

### *Scrapie Inoculum Clearance by Macrophages*

The first clodronate treatment ensured splenic macrophages depletion at the day of scrapie infection (Fig. 4a). The consequences of it were that (i) more PrPres associated with the scrapie inoculum was detectable in the spleen the day of infection and (ii) there was an increase in newly synthesized PrPres during 20 days post-inoculation (Fig. 4a). Thus, macrophage depletion induced prior to scrapie inoculation increased the amount of detectable inoculum in the spleen the day of infection. This excess of inoculum consequently induced a transient increased in newly synthesized PrPres, during the early steps of the pathogenesis. This study illustrates the involvement of splenic macrophages in the clearance of scrapie inoculum at the day of infection, most probably resulting from their ability to phagocytose insoluble PrPres.

### *Involvement of Macrophages in PrPres Catabolism*

We next assessed the outcome for PrPres synthesis of macrophage depletion at the end of inoculum clearance phase (Fig. 4b). This regimen also led to a significant increase of spleen PrPres accumulation (Fig. 4b). This suggests that, in untreated mice, macrophages have

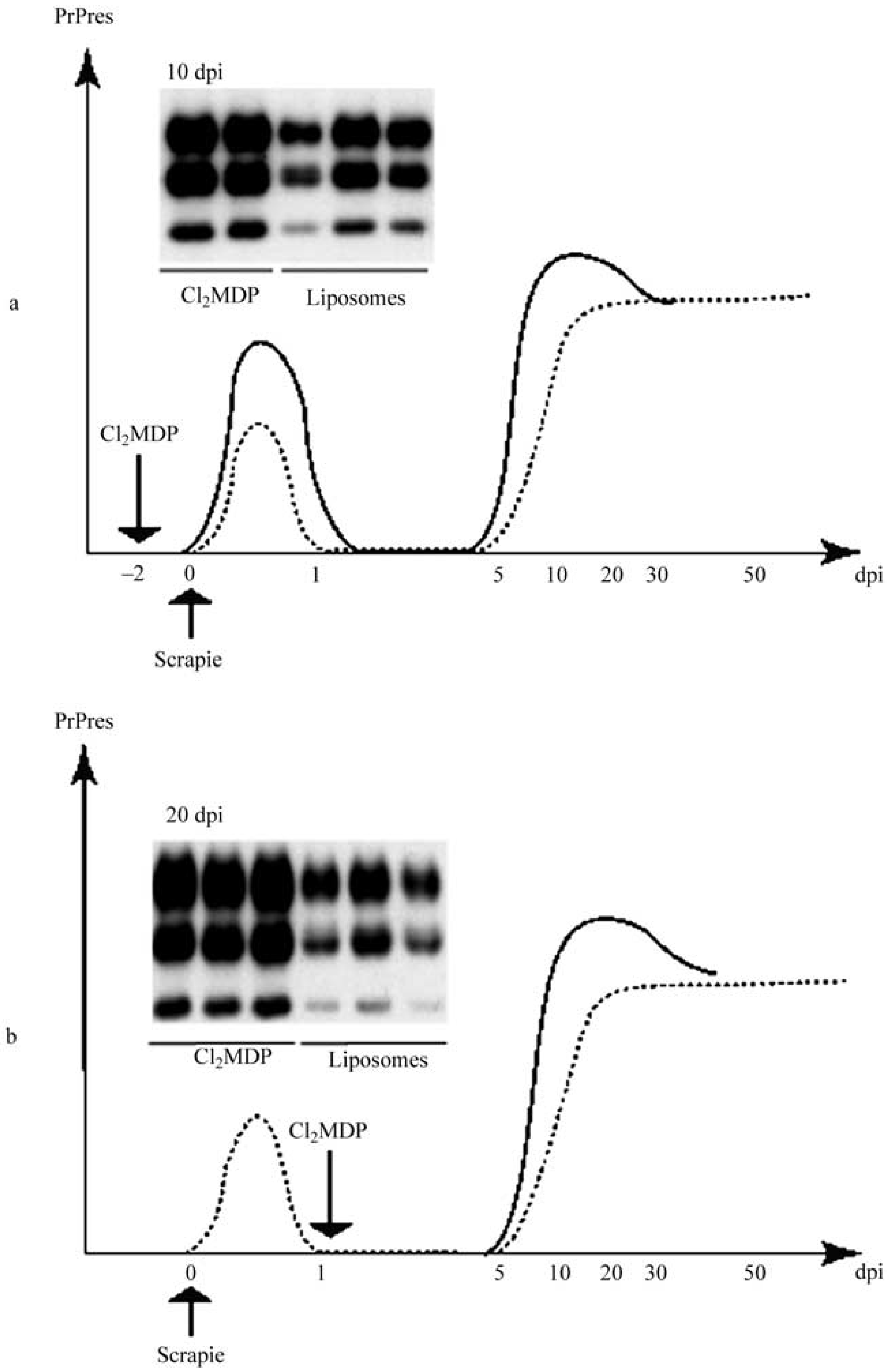

FIGURE 4 PrPres accumulation in the spleen of scrapie-infected mice, following clodronate-containing liposomes administration. Liposome-encapsulated clodronate was injected either 2 days before (a) or 1 day after (b) scrapie inoculation (plain line). Representative spleen PrPres detection is presented at 10 (a) and 20 (b) days post-inoculation (dpi). Empty liposomes-treated mice have been used as controls (dotted line).

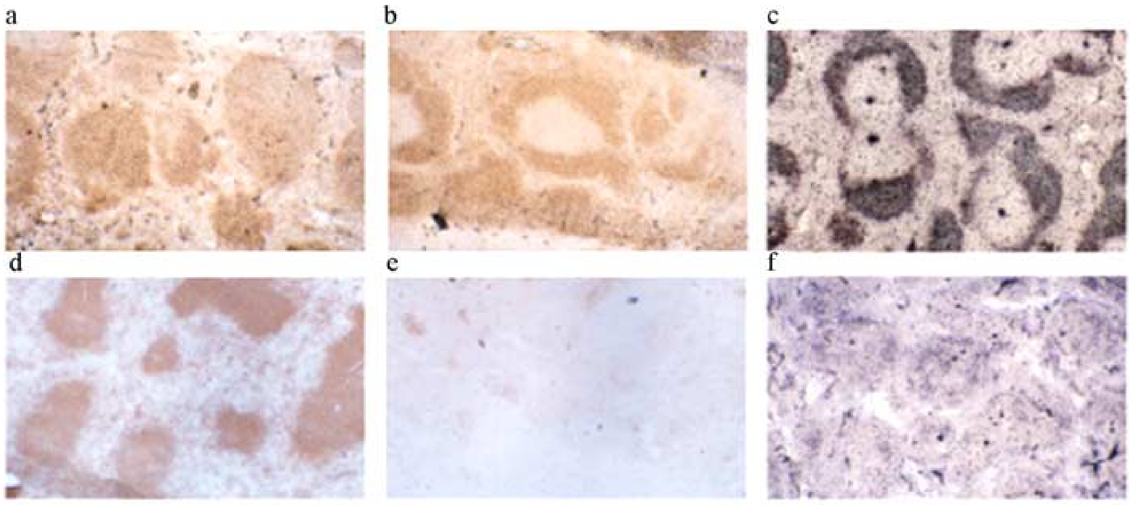

FIGURE 5 Follicular dendritic cells, B lymphocytes but not T lymphocytes are transiently affected by clodronate treatment. Spleen cryostat sections of untreated or empty liposomes-treated (a–c) and clodronate-treated mice (d–f) were prepared. T lymphocytes, B lymphocytes and follicular dendritic cells were immunohistochemically stained with anti-Thy1.2 (a and d), anti-B220 (b and e) and anti-CD35, clone 8C12 (c and f), respectively. Analyses were performed 10 days after liposome-encapsulated clodronate administration. (×40).

the capacity to eliminate newly synthesized PrPres molecules, produced in small amounts just after the post-clearance phase in the spleen (Beringue *et al.*, 1999c).

### *Cells Involved in Scrapie Agent Replication after Clodronate Administration?*

We also studied whether other cells in the spleen were depleted by intravenous clodronate administration, particularly those reported to be involved in TSE pathogenesis. This was necessary to ensure that the effects we observed with clodronate were specific to macrophage depletion. Using standard immunohistochemistry, T lymphocytes were unaffected by the treatment (Fig. 5a and d), as previously described (Laman *et al.*, 1990; Nair *et al.*, 1995). In contrast, B lymphocytes were strongly depleted during the 6–16 day period after $Cl_2MDP$-containing liposomes administration (Fig. 5b and e) but repopulated very rapidly (Beringue *et al.*, 1999c). FDC numbers were also reduced between the 3–16 day period, the strongest effect seen at day 10 (Fig. 5c and f); thereafter the number of FDC slowly increased (Beringue *et al.*, 1999c). These alterations were specific for clodronate administration, as the injection of liposomes alone did not modify immunohistochemical staining of the cell populations studied (Fig. 5a and c). Possibly, FDC and B cells have been reported to be involved in scrapie agent replication in the spleen (Clarke and Kimberlin, 1984; Fraser and Farquhar, 1987; McBride *et al.*, 1992; Muramoto *et al.*, 1993; Klein *et al.*, 1997; 1998). The depletion of these cells after clodronate administration, when detectable PrPres is increased in the spleen suggests the existence of compensatory mechanisms at the cellular or tissue level allowing the scrapie agent to persist and even replicate in the absence of B cells and FDC. As T cells were not affected by clodronate administration, it is tempting to suggest that they have a potential to be target cells. This would occur only in situations where the preferential targets, such as FDC, are not available, as a preponderant role has never been described for T lymphocytes (Fraser and Dickinson, 1978; Mohri *et al.*, 1987; Klein *et al.*, 1997; Raeber *et al.*, 1999a). It is noteworthy that in situations of T and B lymphocytes and FDC impairment, such as in SCID mice, no synthesis of PrPres was detectable in the spleen early in the infection (Beringue *et al.*, 1999a).

### *The Plateau of Spleen PrPres Accumulation may Correspond to a lack of PrPres Synthesis*

A last clodronate treatment was performed when PrPres accumulation, as infectivity (Kimberlin and Walker, 1979) had reached a plateau. The plateau phase has been proposed as the consequence of the restriction of the scrapie agent replication, due to the limited number of non-dividing target cells (Kimberlin and Walker, 1988). But whether the plateau results from a cessation of agent replication when all target cells are saturated, or from a dynamic equilibrium between synthesis and degradation remains to be determined (Kimberlin and Walker, 1988). A transient disappearance of FDC, B cells and macrophages, induced by clodronate treatment during the plateau phase did not modify PrPres accumulation (Beringue *et al.*, 1999c) unlike the earlier treatments (Fig. 4), supporting the idea of a lack of PrPres synthesis and therefore, a cessation of replication activity in the target cells at this time. The absence of effects obtained with clodronate during the plateau is similar to that observed with polyanions, although these drugs have profond effects on the immune system. They could not delay experimental scrapie when administered remote from the time of inoculation, despite being efficient when given around the time of infection (Farquhar and Dickinson, 1986).

### CONCLUSION

Even if the immune cells involved in TSE agent replication and transport are not clearly known, all these data suggest that macrophages can interact and interfere with TSE agent propagation. These effects are probably more prominent in the very early stage of infection in the periphery as late treatment with clodronate did not modify the course of PrPres accumulation in the spleen, and, as the amount of infectivity carried by macrophages did not vary greatly during the time course of scrapie (Table II and Carp *et al.*, 1994). This role of macrophages as the first natural host defender at the time of TSE infection is similar to that observed in conventional diseases (Seiler *et al.*, 1997). The involvement of macrophages also suggests that they could passively contribute to TSE agent propagation, if they are not able to destroy them, due to the strong resistance of PrPres to proteolysis (Prusiner, 1982). Finally, clodronate treatment has indicated the complexity of the involvement and exact role of immunocompetent cells in scrapie pathogenesis. FDC and lymphoid cells might act cooperatively and one population could be able to take the relay of the other one in case of cellular homeostasis modification. This may be an important complicating factor in the ongoing search for the cellular targets of TSEs.

### *Acknowledgements*

We gratefully acknowledge M. Demoy, B. Gouritin, C. Weingarten and J.P. Andreux for their pharmaceutical and immunohistochemical expertise, S. Hawke for careful reading of the manuscript. V. Beringue is a recipient of a fellowship from the Fondation pour la Recherche Medicale (Paris, France).

### *References*

Adjou, K.T., Demaimay, R., Deslys, J.P., Lasmezas, C.I., Beringue, V., Demart, S., Lamoury, F., Seman, M. and Dormont, D. (1999) "MS-8209, a water-soluble amphotericin B derivative, affects both scrapie agent replication and PrPres accumulation in Syrian hamster scrapie", *Journal of General Virology* **80**, 1079–1085.

Beekes, M., Baldauf, E. and Diringer, H. (1996) "Sequential appearance and accumulation of pathognomonic markers in the central nervous system of hamsters orally infected with scrapie", *Journal of General Virology* **77**, 1925–1934.

Beekes, M., McBride, P.A. and Baldauf, E. (1998) "Cerebral targeting indicates vagal spread of infection in hamsters fed with scrapie", *Journal of General Virology* **79**, 601–607.

Beringue, V., Adjou, K.T., Lamoury, F., Maignien, T., Deslys, J.P., Race, R. and Dormont, D. (1999a) "Opposite effects of dextran sulfate 500, the polyene antibiotic MS-8209 and Congo red on accumulation of the protease-resistant isoform of PrP in the spleens of mice inoculated intraperitoneally with the scrapie agent", *Journal of Virology* **74**, 5432–5440.

Beringue, V., Lasmezas, C.I., Adjou, K.T., Demaimay, R., Lamoury, F., Deslys, J.P., Seman, M. and Dormont, D. (1999b) "Inhibiting scrapie neuroinvasion by polyene antibiotic treatment of SCID mice", *Journal of General Virology* **80**, 1873–1877.

Beringue, V., Demoy, M., Lasmezas, C.I., Gouritin, B., Weingarten, C., Deslys, J.P., Andreux, J.P., Couvreur, P. and Dormont, D. (1999c) "Role of spleen macrophages in the clearance of scrapie agent early in the pathogenesis", *Journal of Pathology* **190**, 495–502.

Brown, P., Gibbs, C.J., Rodgers-Johnson, P., Asher, D.M., Sulima, M.P., Bacote, A., Goldfarb, L.G. and Gajdusek, D.C. (1994) "Human spongiform encephalopathy: the National Institute of Health series of 300 cases of experimentally transmitted diseases", *Annals of Neurology* **35**, 513–529.

Carp, R.I. and Callahan, S.M. (1981) "*In vitro* interaction of scrapie agent and mouse peritoneal macrophages", *Intervirology* **16**, 8–13.

Carp, R.I. and Callahan, S.M. (1982) "Effect of mouse peritoneal macrophages on scrapie infectivity during extended *in vitro* incubation", *Intervirology* **17**, 201–207.

Carp, R.I. and Callahan, S.M. (1985) "Effect of prior treatment with thioglycolate on the incubation period of intraperitoneally injected scrapie", *Intervirology* **24**, 170–173.

Carp, R.I., Callahan, S.M., Patrick, B.A. and Mehta, P.D. (1994) "Interaction of scrapie agent and cells of the lymphoreticular system", *Archives of Virology* **136**, 255–268.

Caughey, B. and Chesebro, B. (1997) "Prion protein and the transmissible spongiform encephalopathies", *Trends in Cell Biology* **7**, 56–62.

Clarke, M.C. and Kimberlin, R.H. (1984) "Pathogenesis of mouse scrapie: distribution of agent in the pulp and stroma of infected spleens", *Veterinary Microbiology* **9**, 215–225.

Clayette, P., Martin, M., Beringue, V., Dereuddre-Bosquet, N., Adjou, K.T., Seman, M. and Dormont, D. (1999) "Effects of MS-8209, an amphotericin B derivative on tumor necrosis factor alpha synthesis and human immunodeficiency virus replication in macrophages", *Antimicrobial Agents and Chemotherapy* **44**, 405–407.

Collinge, J. (1999) "Variant Creutzfeldt-Jakob disease", *Lancet* **354**, 317–323.

Dormont, D., Herodin, F., Delasnerie-Laupretre, N., Deslys, J.P., Maurel, C., Chaffanet, M. and Court, L. (1989) "Biochemical and immunological events during unconventional viruses infections: 1. GFAP, IgG, IgM, complement evolutive patterns. 2. Effects of modifications of reticuloendothelial system on scrapie incubation period", In: Court, L.A., Dormont, D., Brown, P. and Kingsbury, D.T., eds, *Unconventional Viruses of the Central Nervous System* (CEA Diffusion, Paris), pp 271–287.

Duval, X., Clayette, P., Dereuddre-Bosquet, N., Fretier, P., Martin, M., Salmon-Ceron, D., Gras, G., Vilde, J.L. and Dormont, D. (1997) "Dapsone and HIV-1 replication in primary cultures of lymphocytes and monocyte-derived macrophages", *AIDS* **11**, 944–945.

Farquhar, C.F. and Dickinson, A.G. (1986) "Prolongation of scrapie incubation period by an injection of dextran sulphate 500 within the month before or after infection", *Journal of General Virology* **67**, 463–473.

Fraser, H. and Dickinson, A.G. (1978) "Studies of the lymphoreticular system in the pathogenesis of scrapie: the role of spleen and thymus", *Journal of Comparative Pathology* **88**, 563–573.

Fraser, H. and Farquhar, C.F. (1987) "Ionising radiation has no influence on scrapie incubation period in mice", *Veterinary Microbiology* **13**, 211–223.

Fu, Y.X., Huang, G., Wang, Y. and Chaplin, D.D. (1998) "B lymphocytes induce the formation of follicular dendritic cell clusters in a lymphotoxin alpha-dependent fashion", *Journal of Experimental Medicine* **187**, 1009–1018.

Gonzalez, M., Mackay, F., Browning, J.L., Kosco-Vilbois, M.H. and Noelle, R.J. (1998) "The sequential role of lymphotoxin and B cells in the development of splenic follicles", *Journal of Experimental Medicine* **187**, 997–1007.

Grathwohl, K.U.D., Horiuchi, M., Ishiguro, N. and Shinagawa, M. (1996) "Improvement of PrPsc-detection in mouse spleen early at the preclinical stage of scrapie with collagenase-completed tissue homogenization and Sarkosyl-NaCl extraction of PrPsc", *Archives of Virology* **141**, 1863–1874.

Greene, L.A. and Tischler, A.S. (1976) "Establishment of a noradrenergic clonal line of rat adrenal phaeochromocyta cells which respond to nerve growth factor", *Proceedings of the National Academy of Sciences USA* **73**, 2424–2428.

Hadlow, W.J., Kennedy, R.C. and Race, R.E. (1982) "Natural infection of Suffolk sheep with scrapie virus", *Journal of Infectious Disease* **146**, 657–664.

Hill, A.F., Butterworth, R.J., Joiner, S., Jackson, G., Rossor, M.N., Thomas, D.J., Frosh, A., Tolley, N., Bell, J.E., Spencer, M., King, A., Al-Sarraj, S., Ironside, J.W., Lantos, P.L. and Collinge, J. (1999) "Investigation of variant Creutzfeldt-Jakob disease and other human prion diseases with tonsil biopsy samples", *Lancet* **353**, 183–189.

Jeffrey, M., McGovern, G., Goodsir, C.M., Brown, K.L. and Bruce, M.E. (2000) "Sites of prion protein accumulation in scrapie-infected mouse spleen revealed by immuno-electron microscopy", *Journal of Pathology* **191**, 323–332.

Kimberlin, R.H. and Walker, C.A. (1979) "Pathogenesis of mouse scrapie: dynamics of agent replication in spleen, spinal cord and brain after infection by different routes", *Journal of Comparative Pathology* **89**, 551–562.

Kimberlin, R.H. and Walker, C.A. (1988) "Pathogenesis of experimental scrapie", *Ciba Foundation Symposium* **135**, 37–62.

Kimberlin, R.H. and Walker, C.A. (1989) "The role of the spleen in the neuroinvasion of scrapie in mice", *Virus Research* **12**, 201–212.

Kimberlin, R.H. and Walker, C.A. (1990) "Intraperitoneal infection with scrapie is established within minutes of injection and is non-specifically enhanced by a variety of different drugs", *Archives of Virology* **112**, 103–114.

Kitamoto, T., Mohri, S. and Tateishi, J. (1989) "Organ distribution of proteinase-resistant protein in humans and mice with Creutzfeldt-Jakob disease", *Journal of General Virology* **70**, 3371–3379.

Klein, M.A., Frigg, R., Flechsig, E., Raeber, A.J., Kalinke, U., Bluethmann, H., Bootz, F., Suter, M., Zinkernagel, R.M. and Aguzzi, A. (1997) "A crucial role for B cells in neuroinvasive scrapie", *Nature* **390**, 687–690.

Klein, M.A., Frigg, R., Raeber, A.J., Flechsig, E., Hegyi, I., Zinkernagel, R.M., Weissmann, C. and Aguzzi, A. (1998) "PrP expression in B lymphocytes is not required for prion neuroinvasion", *Nature Medicine* **4**, 1429–1433.

Laman, J.D., Kors, N., Van Rooijen, N. and Claassen, E. (1990) "Mechanism of follicular trapping: localization of immune complexes and cell remnants after elimination and repopulation of different spleen cell populations", *Immunology* **71**, 57–62.

Lasmezas, C.I., Cesbron, J.Y., Deslys, J.P., Demaimay, R., Adjou, K.T., Rioux, R., Lemaire, C., Locht, C. and Dormont, D. (1996) "Immune system-dependent and -independent replication of the scrapie agent", *Journal of Virology* **70**, 1292–1295.

Lasmezas, C.I., Deslys, J.P., Robain, O., Jaegly, A., Beringue, V., Peyrin, J.M., Fournier, J.G., Hauw, J.J., Rossier, J. and Dormont, D. (1997) "Transmission of the BSE agent to mice in the absence of detectable abnormal prion protein", *Science* **275**, 402–405.

Lavelle, G.C., Sturman, L. and Hadlow, W.J. (1972) "Isolation from mouse spleen of cell populations with high specific infectivity for scrapie virus", *Infection and Immunity* **5**, 319–323.

Maignien, T., Lasmezas, C.I., Beringue, V., Dormont, D. and Deslys, J.P. (1999) "Pathogenesis of oral route infection with scrapie and BSE", *Journal of General Virology* **80**, 3035–3042.

Manuelidis, L., Fritch, W. and Xi, Y.G. (1997) "Evolution of a strain of CJD that induces BSE-like plaques", *Science* **277**, 94–98.

Manuelidis, L., Fritch, W. and Zaitsev, I. (1998) "Dapsone to delay symptoms in Creutzfeldt-Jakob disease", *Lancet* **352**, 456.

McBride, P.A., Eikelenboom, P., Kraal, G., Fraser, H. and Bruce, M.E. (1992) "PrP protein is associated with follicular dendritic cells of spleens and lymph nodes in uninfected and scrapie-infected mice", *Journal of Pathology* **168**, 413–418.

Mohri, S., Hanada, S. and Tateishi, J. (1987) "Lack of effect of thymus and spleen on the incubation period of Creutzfeldt-Jakob disease in mice", *Journal of General Virology* **68**, 1187–1189.

Muramoto, T., Kitamoto, T., Tateishi, J. and Goto, I. (1993) "Accumulation of abnormal prion protein in mice infected with Creutzfeldt-Jakob Disease via intraperitoneal route: a sequential study", *American Journal of Pathology* **143**, 1470–1479.

Nair, S., Buiting, A.M.J., Rouse, R.J.D., Van Rooijen, N., Huang, L. and Rouse, B.T. (1995) "Role of macrophages and dendritic cells in primary cytotoxic T lymphocytes responses", *International Immunology* **7**, 679–688.

Naito, M., Nagai, H., Kawano, S., Umezu, H., Zhu, H., Moriyama, H., Yamamoto, T., Takatsuka, H. and Takey, K. (1996) "Liposome-encapsulated dichloromethylene diphosphonate induces macrophage apoptosis *in vivo* and *in vitro*", *Journal of Leukocyte Biology* **60**, 337–344.

O'Rourke, K.I., Huff, T.P., Leathers, C.W., Robinson, M.M. and Gorham, J.R. (1994) "SCID mouse spleen does not support scrapie agent replication", *Journal of General Virology* **75**, 1511–1514.

Prusiner, S.B. (1982) "Novel proteinaceous infectious particles cause scrapie", *Science* **216**, 136–144.

Prusiner, S.B., Scott, M.R., DeArmond, S.J. and Cohen, F.E. (1998) "Prion protein biology", *Cell* **93**, 337–348.

Raeber, A.J., Sailer, A., Hegyi, I., Klein, M.A., Rulicke, T., Fischer, M., Brandner, S., Aguzzi, A. and Weissmann, C. (1999a) "Ectopic expression of prion protein (PrP) in T lymphocytes or hepatocytes of PrP knockout mice is insufficient to sustain prion replication", *Proceedings of the National Academy of Sciences USA* **96**, 3987–3992.

Raeber, A.J., Klein, M.A., Frigg, R., Flechsig, E., Aguzzi, A. and Weissmann, C. (1999b) "PrP-dependent expression of prions with splenic but not circulating lymphocytes of scrapie-infected mice", *EMBO Journal* **18**, 2702–2706.

Robinson, M.M. and Gorham, J.R. (1990) "Pathogenesis of hamster scrapie. Adherent splenocytes are associated with relatively high levels of infectivity", *Archives of Virology* **112**, 283–289.

Rubenstein, R., Carp, R.I. and Callahan, S.M. (1984) "*In vitro* replication of scrapie agent in a neuronal model: infection of PC12 cells", *Journal of General Virology* **65**, 2191–2198.

Seiler, P., Aichele, P., Odermatt, B., Hengartner, H., Zinkernagel, R.M. and Schwendener, R.A. (1997) "Crucial role of marginal zone macrophages and marginal zone metallophils in the clearance of lymphocytic choriomeningitis virus infection", *European Journal of Immunology* **27**, 2626–2633.

Van Keulen, L.J., Schreuder, B.E., Maloen, R.H., Mooij-Harkes, G., Vromans, M.E. and Langeveld, J.P. (1996) "Immunohistochemical detection of prion protein in lymphoid tissues of sheep with natural scrapie", *Journal of Clinical Microbiology* **34**, 1228–1231.

Van Rooijen, N. (1989) "The liposome-mediated macrophage "suicide" technique", *Journal of Immunological Methods* **124**, 1–6.

Van Rooijen, N. (1991) "High and low cytosolic $Ca^{2+}$ induced macrophage death?", *Cell Calcium* **12**, 381–384.

Van Rooijen, N. and Sanders, A. (1994) "Liposome mediated depletion of macrophages: mechanism of action, preparation of liposomes and applications", *Journal of Immunological Methods* **174**, 83–93.

Van Rooijen, N., Kors, N. and Kraal, G. (1989) "Macrophage subset repopulation in the spleen: differential kinetics after liposome-mediated elimination", *Journal of Leukocyte Biology* **45**, 97–104.

Van Rooijen, N., Kors, N., Ende, M.V.D. and Dijkstra, C.D. (1990) "Depletion and repopulation of macrophages in spleen and liver of rat after intravenous treatment with liposome-encapsulated dichloromethylene diphosphonate", *Cell Tissue Research* **260**, 215–222.

